# Interleukin 2 Topical Cream for Treatment of Diabetic Foot Ulcer: Experiment Protocol

**DOI:** 10.2196/resprot.4036

**Published:** 2015-08-14

**Authors:** Shu Wing Sophia Chan

**Affiliations:** ^1^ currently no institutional affiliation

**Keywords:** type 1 diabetes, topical cream, chronic wound healing, immunotherapy, IL-2

## Abstract

**Background:**

It is estimated there are 2.9 million diabetic patients in the United Kingdom, and around 5%-7% of patients have diabetic ulcers. This number will continue to increase globally. Diabetic ulcers are a major economic burden on the healthcare system. More than £650 million is spent on foot ulcers or amputations each year, and up to 100 people a week have a limb amputated due to diabetes. In T1DM, the level of IL-2 is reduced, and hence, wound healing is in a prolonged inflammatory phase. It is not known if IL-2 topical cream can shorten the healing process in T1DM patients.

**Objective:**

The objective of this study is to understand the pathophysiology in type 1 diabetes (T1DM) and investigate possible future treatment based on its clinical features. The hypothesis is that IL-2 cream can speed up wound healing in NOD mice and that this can be demonstrated in a ten-week study. An experiment protocol is designed in a mouse model for others to conduct the experiment. The discussion is purely based on diabetic conditions; lifestyle influences like smoking and drinking are not considered.

**Methods:**

Skin incisions will be created on 20 nonobese diabetic (NOD) mice, and IL-2 topical cream will be applied in a 10-week study to prove the hypothesis. Mice will be randomly and equally divide into two groups with one being the control group.

**Results:**

T1DM patients have a decreased number of T regulatory (Treg) cells and interleukin 2 (IL-2). These are the keys to the disease progression and delay in wound healing. Diabetic ulcer is a chronic wound and characterized by a prolonged inflammatory phase.

**Conclusions:**

If the experiment is successful, T1DM patients will have an alternative, noninvasive treatment of foot ulcers. In theory, patients with other autoimmune diseases could also use IL-2 topical cream for treatment.

## Introduction

### Background

#### Clinical Problem

The incidence of one major cause of chronic wounds, the diabetic ulcer, is increasing globally. There are an estimated 2.9 million diabetic patients in the United Kingdom; around 5%-7% suffer from diabetic ulcers, and 67% have one or more risk factors. These numbers are expected to increase. Up to 85% of amputations are preceded by foot ulcers, and a recent World Health Organization report pointed out that a reduction in amputations could be achieved through success in reducing ulcer incidence [1]. According to a recent National Health Service report, £650 million is spent on foot ulcers or amputations each year, and up to 100 people a week have a limb amputated due to diabetes [2]. This research proposal is aiming to help patients with type 1 diabetes in wound healing using immunology principles.

#### Immunology in Type 1 Diabetes

Type 1 diabetes (T1DM) is an autoimmune disease the cause of which remains unknown. In T1DM patients, β-cells in the pancreas are destroyed by autoimmune responses, resulting in a lack of insulin production. Scientists generally believe that it arises either by genetic or environmental factors, and those factors contribute three criteria for developing T1DM. First, β-cell-reactive T cells are being activated, and hence, a proinflammatory response is induced. Last and most importantly, the immune regulation of autoreactive responses fails [3].

#### Environmental Factors

The enterovirus is believed to be the most common viral infection causing T1DM. Other viruses like rotavirus, mumps, cytomegalovirus, and Epstein-Barr virus might be able to trigger autoimmune responses or associate with T1DM, as they have the ability to infect or lyse β-cells directly [4]. During infection, the number of pattern recognition receptors increases in the islets to facilitate the innate immune system’s identification of microorganisms. The virus activates the production of interferon α (IFN-α) and IFN-β chemokines and overexpresses the major histocompatibility complex (MHC) I molecule; subsequently, the chemokines attract T cells to produce proinflammatory cytokines such as interleukin 1 β (IL-1β) and IFN-γ. As the infection process continues, antibodies and cytotoxic T lymphocytes disrupt β-cells and induce apoptosis. Killing is mediated via the interaction between Fas ligands on the CD4^+^ T cell and Fas on the β-cell, and eventually the β-cell antigen (MHC II molecule) will be released.

The reason why the infection is chronic is that specific antibodies from persistent infections can target Coxsackievirus (one of the RNA enteroviruses) and adenovirus receptors and FcγRII and III to enhance the infection of monocytes and macrophages. Antibody-dependent enhancement of infected monocytes and macrophages could increase the spread of the virus to β-cells and continuously stimulate autoimmunity.

Initial viral infection in children is thought to be beneficial, as their bodies develop adaptive immunity. However, in T1DM patients, T regulatory (T_reg_) cells are reduced due to an ineffective regulatory system. Hence, viruses keep replicating and persistently result in high concentrations.

#### Human Genetic Factors

Genetic factors have a big impact on the abnormal immune system in T1DM patients. They are blamed for about one-third of the susceptibility to T1DM, and over 20 different regions report such linkage [4]. Viral infection may trigger autoimmunity, but it is only part of the disease mechanism. Apart from activating the different kinds of immune cells, the body also synthesizes different enzymes in response to the invasion, and genes control this. Gene *IDDM10* has been picked as an example here.

The IL-2 receptor is expressed on immune cells in response to the stimulation of T-cell receptors (TCRs) during antigen binding. This could increase the binding of IL-2, which is vital in T-cell proliferation. The IL2α chain (CD25) is part of the IL-2 receptor and expresses during the predevelopment stage of the T and B lymphocytes. Chromosome 10 contains more than one susceptibility locus; they are called IDDM10. One of the regions is 10p15-p14, where the IL2α chain (CD25) is encoded. Mutation in this region would possibly lead to an IL-2 receptor α deficiency and hence affect the FOXP3 protein. Tag single-nucleotide polymorphisms were analysed, and it was discovered that the deficiency is likely due to linkage disequilibrium [5]. Interestingly, FOXP3 is strongly associated with T1DM; however, its gene (on chromosome Xp11) has no genetic association in T1DM patients [6].

#### Immune Response in Type 1 Diabetes

Various T cells are activated when MHC I and II are expressed by a virus or β-cell. At the same time, exposure of proinflammatory cytokines on a β-cell drives the β-cell to upregulate IL-8 and chemokine (c-c motif) ligand 5 (CCL5), which draw target cells to migrate to the infection site by chemotaxis. In normal practice, CD4^+^ T cells express IL-22, while islet cells express the IL-22 receptor to activate the signal transducer and activator of transcription (STAT) 3 [3]. STAT3 is important to compete with IL-2 and hence to upregulate protective gene transcription. The difference between a healthy individual and a diabetic patient is the introduction of IFN-α, which makes IL-22 switch STAT3 to STAT1. STAT1 is responsible for the expression of inducible nitric oxide synthetase.

T_reg_ cells are the critical immune cells in T1DM immune modulation. These cells are important, as they monitor and kill autoreactive T cells to prevent pathological self-reactivity. In patients with T1DM, T_reg_ cells no longer effectively control the islet autoreactive T cells and consequently, the body loses immune tolerance and continuously activates B cells and effector T cells. However, the nature of the dysfunction remains unclear [7].

FOXP3 is a transcriptional factor which acts as lineage specification factor of T_reg_ cells. Its specific contribution in the differentiation and function of T_reg_ cells remains uncertain. Several experiments have been done in animals and humans to test the effects of a defective FOXP3 or a deficiency in FOXP3. Evidence shows that FOXP3 is critical in maintaining self-tolerance by suppressing self-reactive T cells. One may expect that the faulty FOXP3 gene is crucial in contributing to T1DM. It is true that patients with FOXP3 deficiency will develop immunodysregulation polyendocrinopathy enteropathy X-linked syndrome (IPEX). More than 80% of IPEX patients acquire T1DM at very early onset [8]. Indeed, *IDDM10*, which encodes the IL2α chain (CD25), is the causative reason. CD25 is so important because it is the key cytokine for the fitness and function of FOXP3^+^ T_reg_ cells. In other words, CD25 maintains the stability of and upregulates FOXP3. It is believed that it involves a direct pathway linking IL-2 signalling to the expression of the FOXP3 gene through STAT proteins [9]. Studies [10] also show that IL-2 is vital for T_reg_ cell survival. In T1DM patients levels of IL-2 are reduced, and one can deduce that this will affect FOXP3 effects on T_reg_ cells. It is believed that the loss of expression of FOXP3 not only results in a loss of regulatory function but could also be associated with the conversion of T_reg_ cells into potential effector T cells and with the secretion of IFN-γ and IL-17. These cytokines may further reduce the secretion of IL-2 and form a vicious circle. Another suggestion is that the effector could become resistant to suppression [7]. The elevated amount of T_H_1 and T_H_17 is the evidence of failure of immune suppression. The numbers increase throughout T1DM’s development.

Cytotoxic T-lymphocyte–associated protein 4 (CTLA-4) is a protein receptor on the T-cell surface that downregulates possibly by recruiting phosphatase to the TCR. There is controversy about the main functions of CTLA-4; it could be involved in another essential part of the T_reg_ cell function but is perhaps not involved in the expression of FOXP3 [11]. Serum levels of sCTLA-4 (soluble form) are higher in patients with autoimmune thyroid diseases compared to healthy subjects. However, it is not clear whether this phenomenon is due to DNA mutations or to an indirect effect correlated to the affection status [12]. The reason for functional change in immune regulation caused by mutation is unknown. T-cell development is not affected in T1DM patients but the number and activities of T_reg_ cells appear unusual. Investigation shows that performance of suppression by T_reg_ cells is weaker in the higher ratio of effector T cells [13]. Different evidence demonstrates that T_reg_ cell functions are impaired in patients with T1DM. Perhaps this affects the wound-healing processes.

#### Immunology at the Cellular Level

Although the T1DM pathophysiology is different from T2DM, both types of diabetes share many clinical features (eg, insulin resistance, cardiovascular disorders). The mechanism is not fully discovered yet; a paper suggested insulin therapy might be a primary inducer. In a cultured hepatocytes model, significant insulin resistance was developed under prolonged exposure to insulin, and this is similar to the setting of insulin therapy [14]. Apart from insulin resistance, high triglyceride level is often measured in patients with persistent or inadequate insulin therapy. On the other hand, untreated T1DM patients may not face the problem of insulin resistance [15]; however, hyperglycemia could lead to ketoacidosis. Ketoacidosis eventually enhances the flux of free fatty acid (FFA) to the liver and promotes hypertriglyceridemia [16]. In addition, in obese subjects with T1DM, insulin resistance can accelerate progression of T1DM complications [14].

It is believed that there are inflammatory events around adipose tissue and immune responses to elevated blood glucose. Those events can be observed in all tissues that process energy homeostasis (eg, fat, muscle, liver, and blood vessels) [17]. Eventually, once these factors are transported to the pancreas by the blood, islet autoimmunity might occur [18]. FFA interacts with toll-like receptors (TLRs) and facilitates the following immune activities. First, FFAs induce the formation of lipid rafts in cell membranes, which favor the TLRs’ signalling. Production of reactive oxygen species (ROS) is involved in the activation of inflammatory pathways via TLR. Hence, ROS may lead to the induction of stress kinases. ROS could also initiate the IL-1 system through the formation of NLRP3 inflammasomes. Meanwhile, production of IL-1β could be facilitated through FFA metabolites or via TLR2 or 4. FFA can directly activate these receptors, as they are sensitive to lipids [19].

Adipose tissues per se can induce immune responses. Although the mechanism that initiates the inflammation is unknown, inflammation is associated with an accumulation of immune cells in obese patients. It is deduced that hypertrophy is responsible, as cellular contents might leak into cellular spaces and promote an inflammatory response or induce adipocyte death [17]. Earlier research [20] concluded that obesity is associated with an increased accumulation of T cells and macrophages in adipose tissue, and CCL5 is an adipokine that is upregulated in adipose tissue through obesity in humans. However, it is not clear what antigen attracts T cells. Although more T cells are presented in comparison to a leaner state, the ratio between T_reg_ cells and CD4^+^ T cells dramatically decreases with increasing levels of obesity [21]. The impact of T cells in adipose tissue on neighboring cells is likely through soluble mediators. Fat-resident regulatory and conventional T cells can synthesize various cytokines, which have a direct impact on the synthesis of inflammatory mediators and glucose uptake by adipocytes. T_reg_ cells are not just involved in immune response; they could also increase the circulation of blood glucose.

Increased tumor necrosis factor α (TNF-α) has been detected in the adipose tissue of obese mice and is recognized as one of the immune characteristics [17]. This has also been observed in the serum samples of diabetic patients using the enzyme-linked immunosorbent assay [22]. In vitro, TNF-α induces changes in the expression of a number of transcripts that encode inflammatory mediators, including IL-6, CCL5, serum amyloid 3, intracellular adhesion molecule 1 and matrix metallopeptidase (MMP) 3. These cytokines are described as a network that stimulates the production of acute-phase proteins. IL-6 is the main stimulator, while IL-1 and TNF-α are the central mediators of inflammatory reactions [22]. Hypoxia, often seen in hypertrophy, could also raise immune response. Hypoxia can recruit macrophages and induce the expression of numerous proangiogenic and proinflammatory genes inside macrophages. This may be closely associated with infection and the wound-healing process, as the recruitment of macrophages is the key in those events.

Meanwhile, hyperglycemia provokes a similar immune response and activates different cytokines, transcriptional factors, and ROS in the islets. Glucose can stimulate nonenzymatic glycation and generate advanced glycation end products (AGEs), one of the factors in the stimulation of the pattern recognition receptor, RAGE. Once RAGE is activated, the production of nuclear factor-κB (NF-κB), ERK1, ERK2 (MAPK) and ROS will be induced [17]. All these products are fundamental for the next step in immune response—the production of IL-1β and other cytokines. The other important reason to maintain low or normal concentrations of ROS is because β-cells have limited antioxidative enzymes and consequently are susceptible to oxidative stress [19]. Production of mature IL-1β highly depends on caspase 1, as it involves a rate limiting step in IL-1β processing [23]. Caspase 1 plays its role by activating an inflammasome, and eventually the caspase 1 inflammasome secretes mature IL-1β. Apart from ROS, islet amyloid polypeptide (IAPP) contributes to the induction of IL-1β by triggering NLRP3 inflammasome. In addition, stimulation from TLR2, TLR4 and RAGE could lead to NF-κB activation and could produce various cytokines and chemokines, including IL-1β.

Thus far, few activation mechanisms of IL-1β have been mentioned; these are induction via TLR, IAPP, and ROS. In addition to induction, IL-1β could be upregulated under high glucose concentration, as more Fas is likely to be expressed on β-cells, and Fas could induce such secretion.

IL-1 acts as a sensor of metabolic stress and activates immune response in β-cells, insulin-sensitive tissue, and blood vessels [17]. In low concentrations of IL-1β, it can promote β-cell function and survival. However, if IL-1β is continuously activated, a broad range of CC-chemokine ligand will keep being produced, for example, CCL2, CCL3, and CCL8. These are formed via NF-κB activation in epithelial, endothelial, and immunocompetent cells [23]. Those chemokines are known to have a role in monocyte recruitment. At the same time, IL-1β stimulates the IL-1 receptor (IL-1R) on fat cells and later, FFA is liberated. As more and more FFAs are present in the blood, they form a vicious circle of inflammation. IL-1 has been found to have profound effects on the function of β-cells, inducing them to undergo apoptosis via ERK [24]. In brief, IL-1β indirectly recruits macrophages to adipose tissue and islets and amplifies islet inflammation [18]. IL-1 receptor antagonists (IL-1RAs) are greatly expressed in prediabetes; however, the expression decreases along the pathogenesis. This results in an imbalance between the agonist IL-1β and IL-1RA. This indicates that the inflammation might be greatly enhanced due to the susceptibility of β-cells to IL-1β [19].

### Diabetes and Complications: Nephropathy, Atherogenesis, and Neuropathy

#### Diabetic Nephropathy

Diabetic nephropathy is a typical complication in both types of diabetes. It is a progressive and chronic kidney disease (CKD) in the glomerulus and is characterized by hyperfiltration [25]. Microalbuminuria is gradually developed due to the extracellular matrix accumulation in basement membranes and mesangium [26]. CKD does not merely affect the kidney but also impacts the vascular system, immune system, and skin and growth factors [27]. The precise cause of diabetic nephropathy is undiscovered, but scientists suggest that hyperglycemia, activation of cytokines, and inflammation are possible causes [28].

An experimental wound model has been applied in mice and studied with different analyses. It included study of the kidney glomerular tuft area, histology and immunofluorescence, tissue gnostics quantification, and polymerase chain reaction [27]. Although the study was based on CKD, its result helped to widen our understanding. The results verified that CKD wounds had statistically tremendous disruption of normal reepithelialization kinetics and granulation tissue deposition rates. Consequently, the epithelial gap was relatively larger, and the formation of granulation tissue was decreased. In terms of cellular proliferation and angiogenesis, these activities were notably decreased in the early stages of wound healing. At the same time, the inflammatory state was maintained and increased for 14 days. Surprisingly, this research showed that there was no correlation between IL-1β, TNF-α, and wound healing [27]. This might reflect the idea that IL-1β and TNF-α are important in the progression of diabetes but not in wounds.

The physical conditions of wounds have also been reviewed. Dryness, rashes, microangiopathy, and even calciphylaxis were considered to correlate with the severity and duration of the CKD state [27]. Dryness is often associated with infection; however, no wound infections were observed in this experimental setting, and indications were that the wounds were systemically based. Although bacterial infection is not the cause of CKD, infections tend to be observed in patients with CKD as well as those with diabetes-related foot infections. In 653 samples from 379 patients, 23% of wounds were detected with methicillin-resistant *Staphylococcus aureus*; it is believed that the infection is responsible for increased morbidity and mortality [29]. Infections often compromise wound-healing progress.

#### Atherosclerosis

Atherogenesis is considered a threat to healing by contributing prolonged inflammation and inadequate blood and oxygen supply to tissue. Although it alone does not cause ulceration, it can be serious enough to cause amputation of affected limbs [30]. Its characteristics include abnormalities in endothelial cells and in the function of vascular smooth muscle cells and platelets. These abnormalities could induce endothelial dysfunction, an early, integral component of atherosclerosis. The following section studies the connection between diabetes and atherosclerosis. Based on current understanding, inflammation is the key that drives atherosclerosis, and diabetic conditions may in turn promote vascular inflammation.

Nitric oxide (NO) has an imperative role in protecting blood vessels by regulating vasodilation, as well as mediating molecular signals and thus preventing leukocyte and platelet interaction. However, in diabetic patients, hyperglycemia, FFA, and insulin resistance are believed to be the major cause of reduced level of NO. Hyperglycemia and FFA can increase the generation of ROS and hence ROS inactivates NO by disturbing the phosphatidylinositol 3 kinase pathway via the activation of protein kinase C. Meanwhile, FFA could further weaken endothelial function by reducing endothelium-dependent vasodilation. When the liver deals with excess FFA, it enriches production of very-low-density lipoprotein. Plaque is easily formed in diabetic patients because plasma coagulation factors and lesion-based coagulants are elevated, whereas thrombomodulin and protein C decline. A reduced level of NO and endothelial dysfunction favor the migration of vascular smooth muscle cells into nascent atherosclerotic lesions.

Although the individual role of C-reactive protein (CRP) in atherogenesis is still under investigation, it could contribute to atherogenesis together with LDL. It is reported that the CRP level is elevated during inflammation and under hyperlipidemic conditions, and it presents throughout all stages and colocalizes in activated complements in humans [31]. CRP is potentially able to activate macrophages, endothelial cells, and vascular smooth muscle cells, hence promoting inflammation. In addition, CRP could also increase MMP synthesis, as proven by fluorescence microscopy of the endothelial layer. MMPs have been considered the central pathway linking inflammation and plaque instability or rupture [32].

#### Diabetic Neuropathy

Diabetic neuropathy is the most common diabetic complication in both T1DM and T2DM, with 45%-60% of ulcerations believed to be purely neuropathic [1]. It is considered a disorder and contributes by polyol pathway, microvascular injury, and AGEs. Diabetic neuropathy is further classified as peripheral, autonomic, proximal, and focal neuropathy. The following discussion focuses on diabetic peripheral neuropathy (DPN), as it is the most common among all types and is closely linked to the wound-healing processes. The predominant pathological characteristics of DPN are loss of axon, demyelination [30], capillary basement membrane thickening, endothelial cell hyperplasia, and neuronal ischemia and infarction [33]. Large myelinated fibers are generally lost due to toxic and metabolic disorders, yet small unmyelinated fiber losses are also recorded in peripheral nerves. The second characteristic, demyelination, would result in sensitive changes and a decrease in conduction velocity. Notably, this could affect all types of nerves in the central and peripheral nervous systems. Peripheral nerves are surrounded by large numbers of blood vessels to ensure a good blood supply. However, atherogenesis limits blood flow, as the presence of plaque reduces the blood vessel lumen. Insufficient blood flow results in a reduction of nerve conduction velocity as well as circulation of nerve growth factor.

Damaged nerves to intrinsic foot muscles lead to an inequity between flexion and extension of the affected foot. Skin breakdown and ulceration are progressively shown, since foot deformities cause abnormal bony prominences and build pressure points. In addition, neuropathy reduces the functionality of sweat and oil glands. As a result, the foot loses the natural ability to moisturize the skin and hence becomes dry. Often, dry skin increases susceptibility to microorganisms and subsequently to the developing of infection. Patients with severe peripheral neuropathy may also suffer from neuropathic edema in the lower legs. This is possibly related to vasomotor changes and arteriovenous shunting [34]. It is generally believed that the loss of sensation is the biggest problem caused by neuropathy. Patients are often unable to feel pain from the affected site, and thus wounds gradually get worse without being noticed [35].

### Wound Healing Process

#### Origins of Ulcers

Diabetic neuropathic ulcers are most likely to be seen on a patient’s palm and the soles of the feet. In general, a diabetic foot ulcer is more predominant. Ulcers do not happen spontaneously; they can begin with minor skin infections or cuts. Wound healing is a complex process involving blood clotting, inflammation, proliferation, tissue remodelling, and eventually wound closure; the phases overlap each other. Diabetic wounds are chronic wounds in which the wounds are trapped in the inflammation phase and do not heal within three months [36].

#### Inflammatory Phase

Wound healing is a complicated process and involves the nervous and immune systems. The first stage, the inflammatory phase, begins with a passive leakage of circulating leukocytes from damaged blood vessels towards the wound site. T cells and Langerhans cells secrete cytokines and chemokines in response to the injury [37]. Various cell types secrete growth factors, which have a role in recruiting neutrophils and macrophages from nearby uninjured blood vessels. The transforming of growth factor β 1 and vascular endothelial growth factor expression are generally interrupted due to reduced IL-1RA. As mentioned earlier, the level of IL-1RA is reduced in diabetic patients. Reduction could induce prolonged NF-κB translocation, and subsequently lead to suspended wound healing [36]. Cytokines, chemokines, and growth factors all together are the important mediators, and activate intracellular signalling to drive cell proliferation, migration, and differentiation.

Like other chronic wounds, a diabetic ulcer is characterized by the imbalance of proinflammatory and anti-inflammatory factors. This feature possibly arises due to macrophage dysfunction. In diabetic mice, macrophages isolated from a wound showed momentous impairment in efferocytosis; in other words, cell debris was not cleared up effectively, resulting in an accumulation of apoptotic cells. Fas ligands drove the apoptotic cell burden to enhance proinflammatory but weaken anti-inflammatory cytokine response [38]. Although disruption of the blood supply and hypoxia can increase the presence of macrophages at a wound site, it may not benefit healing when the impairment in efferocytosis is considered as well as the delayed migration [39].

#### Proliferative Phase

There are three important events in the proliferative phase: angiogenesis, formation of granulation tissue and the extracellular matrix (ECM), and reepithelialization. Angiogenesis is the most critical of these events and is the key process of successful wound healing. New blood vessels are essential to deliver nutrients and oxygen to support the growth of cells and tissue and the formation of a wound matrix [37].

Cellular hypoxia, a major issue in chronic wounds, is one of the consequences when angiogenesis is blocked or retarded. It is thought that angiogenesis could be affected by diabetic neuropathy to some degree. Various cells need oxygen to survive and function. For example, fibroblasts and epithelial cells require oxygen to migrate, and collagen fibril crosslinking requires oxygen to carry out hydroxylation. Infection is likely to occur with insufficient oxygen, as the bactericidal potency of leukocyte oxidative phosphorylation is changed.

#### Granulation and Reepithelialization

Formation of new granulation tissue and reepithelialization are the mechanisms to restore the function of the skin and bring about permanent closure of the wound gap. Granulation tissue is formed in the dermis, performed by fibroblasts, whereas reepithelialization is carried out in the epidermis by keratinocytes. Both mechanisms correlate with each other closely to bring about the outcome.

Reepithelialization begins with the migration of keratinocytes. Upon arrival at the injured site, keratinocytes modify or dissolve cell-cell and cell-matrix adhesions by releasing collagenases (MMP1) and different types of MMPs. These changes facilitate the formation of a laminin V and collagen IV-rich basement membrane through provisional matrix substrates. Debris and blood clots are also dissolved during this progression to benefit the migration of other cells. Keratinocytes themselves produce growth factors and basement membrane proteins to accelerate reepithelialization [37]. ECM is not just a supportive three-dimensional structure; it also encourages cell proliferation, survival, function, migration, and differentiation. Cell-matrix interaction can indicate the process of ECM remodelling in wound healing [40].

Normal fibroblasts come from different parts of the body and migrate to the wound site. The primary source is the surrounding healthy dermis, while bone marrow progenitor cells and circulated fibrocytes are alternative sources. These cells migrate and join together in response to stress fibers and various growth factors promoting the migration. Fibroblasts use the fibrin crosslinking fibers, formed in the later period of the inflammatory phase, to migrate across the wound, and eventually they adhere to fibronectin. Fibroblasts synthesize, bind, and align collagen fiber into the wound bed in the contraction step [37]. Contraction is the other key step in granulation and is performed by myofibroblasts. Myofibroblasts are differentiated from fibroblasts and are similar to smooth muscle cells in terms of structural and biochemical properties. They express microfilaments and α-smooth muscle actin to connect various wound edges by attaching to desmosomes [41]. Insulin-like growth factor 1 (IGF-1), which is produced by epidermal T cells, is the growth factor that stimulates this process. Experiments on rats have demonstrated that an increase in IGF-1 could enhance the expression of myofibroblasts and tissue repair capacity [41]. Research results showed that a low IGF-1 level is associated with both types of diabetes and, generally, with adults under 65 [42]. Other research on human acute and chronic wounds both in vivo and in vitro also reported there was lower IGF-1 production in chronic wounds under flow cytometry analysis [43]. Scientists proposed that T cells in chronic wounds are less alert to activation and bypass TCR stimulation, likely due to much less IL-2 production by the T cell [43].

There are a few difficulties to consider in the proliferative phase. First, MMPs are excessively synthesized in diabetic patients; the high level of activity could cause unnecessary collagen loss. A high level of MMP1 is vital in wound healing; however, too much MMP8 and 9 could be dangerous. A longitudinal study was conducted on T2DM patients with foot ulcers investigating the impact of MMP levels on recovery. The study recorded levels of MMPs and tissue inhibitors of metalloproteinases around the wound edge and concluded that reduced MMPs do not contribute to good healing [43]. The second issue is glycation of collagen under elevated blood glucose levels. Collagen and elastin could easily carry out irreversible crosslinking with AGEs via nonenzymatic glycation [40]. Evidence proves that both mechanical and biochemical roles are impaired after glycation. The same study pointed out that cell-induced material contraction is also inhibited under observation in immunostaining [44]. In addition, the formation of collagen is retarded in an insufficient oxygen environment.

### Hypothesis and Aims

Impaired function and a decrease in numbers of T_reg_ cells have been discussed in detail above. In T1DM, the level of IL-2 is reduced, and hence, wound healing is in a prolonged inflammatory phase. If optimum IL-2 dosage is given to patients, perhaps it can increase the T_reg_ cell survival rate and response; this likely can be achieved through immunotherapy. Immunotherapy is often used on cancer patients, and research on treating patients with T1DM is ongoing. In 2013, experimental results showed that a low IL-2 dose could increase the number of T_reg_ cells; however, a high dosage might induce β-cell impairment in humans. IL-2 was given by injection in the experiment, and some T1DM patients had serious injection site reactions [45]. Similar results have also been observed in mice. In the nonobese diabetic (NOD) mouse model, mice were given a low dose of IL-2 (25,000 IU per day) for five days, and IL-2 appeared to act specifically on T_reg_ cells in inflamed nonlymphoid tissue. Although the effect was more remarkable in tissue with ongoing autoimmunity such as the pancreas, results also showed that IL-2 can upregulate CD25 expression and suppress IFN-γ in skin. Moreover, no harmful effects were detected in other cells, which means that the risk of generalized immune suppression is low. These two experiments demonstrated that IL-2 could be a safe therapeutic choice for treating inflammation. In terms of the IL-2 effects on wounds, experiments have been done on Adriamycin-treated [46] and Lewis [47] mice and have demonstrated positive effects of IL-2 on wound healing. These experimental results imply that there is hope for IL-2 treatment in healing. The effect of IL-2 on diabetic wounds has not been investigated on animal models or humans. It is predicted IL-2 treatment would only work in T1DM, as there is no reduction of IL-2 in T2DM.

The hypothesis is that IL-2 cream can speed up wound healing in NOD mice and that this can be demonstrated in a ten-week study. Inspired by the success of insulin cream, it is decided that IL-2 will be given in cream form at a low dosage. A human IL-2 gene could be modified by genetic technology and will subsequently be inserted into *Escherichia coli* for synthesis. The first aim is to study the effect of IL-2 cream on the wound-healing process to prove the hypothesis. The second aim is to test the cream’s pharmacokinetics and determine the effective dosage (with no serious side effects).

## Methods

### Intervention

**Figure 1:**
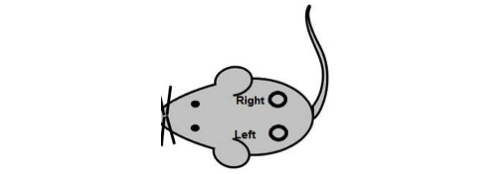
NOD mouse surgery position.

A ten-week-old male NOD mouse’s hair will be removed before the surgery. During the surgery, the mouse will be anesthetized by open-mask method and circular dorsal skin incisions at least 1 cm in diameter will be created on the left- and right-hand sides in parallel (see Figure 1). The left side will be cut through the epidermis and above the dermis, and the right side will be cut through the dermis but not reaching to muscle.

An aseptic technique will be used to prevent bacterial infection. After surgery, a thin protective dressing will immediately be placed on the wound site. Throughout the experiment, all mice will be fed the same standard mouse diet and water and will live in a pathogen-free and temperature-controlled animal facility with a 12-hour light and dark cycle until sacrificed. Mice will be euthanized 24 hours after the last treatment.

Twenty male mice will be randomly and equally divided into two groups. In one group, a thin layer (0.1 g) of IL-2 cream will be applied on each wound every 24 hours; the other group will be the control group and will receive typical wound care. The cream will first be applied 24 hours after the surgery. Both groups will have typical wound care, which includes changing dressings and cleaning the wounds daily with saline water.

As in a previous study, recombinant human IL-2 will be given in a 25,000 IU dosage per day [48] for the first seven days, with future doses being adjusted after evaluation. Active ingredients of the topical cream include 250,000 IU/g recombinant human IL-2, liquid paraffin 6% w/w and white soft paraffin 15% w/w; inactive ingredients are purified water and glycerol.

### Data Collection

Both groups will have the following measurements unless otherwise specified. Two blood samples, a total of 0.3 mL, will be taken from the tail vein before surgery and every 24 hours after daily care. One of the samples will be used to check the level of insulin (pmol/L) in the serum and the white blood cells (K/uL) and the differential of white blood cells, based on 100 cells counted in plasma. Cytokines in serum will specifically be detected by cytokine array for quantitative measurement and will be observed for changes in the amount. T_reg_ cells will be isolated by magnetic cell separation and analysed by fluorescence-activated cell sorting, chosen for studying the surface expression of CD4^+^ and CD25^+^ of the T_reg_ cells [48]. A wound biopsy will be performed every week, starting from day 0, for histologic analysis. The epithelial gap and granulation tissue will be measured after hematoxylin and eosin (H&E) staining under a digital microscope. The percentage of epithelial gap closure and granulation tissue area will be quantified and calculated using a computer image analysis system and pixel density. The epithelial gap is defined as the distance between the edges of keratinocyte migration across the wound [27]. Maximal wound breaking strength will be measured by tensiometer in g/mm^2^ after the mice are sacrificed [49].

Body weight will be measured daily in grams by an electronic balance. A pharmacokinetic test will be conducted by high-pressure liquid chromatography to measure the concentration of IL-2 in plasma in IU/mL [50].

### Statistical Analysis and Expected Results

A 2-tailed unpaired Student *t* test with a 95% confidence interval will be calculated to demonstrate the effect of IL-2 cream in both shallow and deep wounds as well as the difference between the two groups.

The number of immune cells and cytokine trends is expected to be similar to that in normal wound-healing progress. The IL-2 level should reach its peak after 24 hours of administration, while the pharmacokinetics are unknown and should be investigated. It is hoped that the insulin level will increase as well. H&E staining should show a shorter epithelial gap and granulation tissue, and the total epithelial closure of the wound should be bigger in contrast to the control group.

### Ethical Issues

To limit the suffering from the incisions as much as possible, the mice will be given anesthesia before surgery, and the surgeries will be completed within 15 minutes. A mortality risk exists, as this is an invasive operation, but the risk should be low because the surgery does not involve injury to vital organs. Moreover, proper wound care will be given to minimize wound deterioration and infection. All mice in this experiment will receive humane care throughout the study and euthanasia at the end of the study.

IL-2 has been proven effective in cancer patients and has become one of the current treatments. One can see that there is a therapeutic value in conducting a similar experiment. The experiment is unlikely to be replaced by computer models or conducted on humans at this stage, so mouse models are chosen. Dosages will be given in a safety range based on similar experiments and may be adjusted after weekly evaluation of health and progress.

As this is a pilot experiment and no previous data exists, only small numbers of mice are used. The sample size should be big enough to show the differences and prove the hypothesis. Male mice are studied to prevent unnoticed pregnancy in female mice.

## Results

This project is currently on hold as funding has not been secured yet.

## Discussion

Lifestyle changes or surgical intervention could significantly improve ulcers in T2DM patients. However, clinical procedures might not be as effective on T1DM patients. Considering the success of imiquimod cream for treating melanoma and the latest results of IL-2 therapy in diabetic patients, it is realistic to develop topical immunotherapy. So far, no related experiment has been done on animal models or humans. If this experiment is successful, T1DM patients will have an alternative, noninvasive treatment. Since the use of topical cream doesn’t require much education, it will be suitable for both young and elderly patients and will enhance compliance. In addition, the risk of infection and serious skin reaction could be reduced in comparison to intravenous treatment. It is assumed that this treatment can be safely used without contraindications with oral medicine. Last, it can protect skin from dryness as other creams do and can maintain a moist environment for the wound. In theory, patients with other autoimmune diseases could also use IL-2 topical cream for treatment.

## Abbreviations

AGE: advanced glycation end product
CCL: chemokine (c-c motif) ligand
CKD: chronic kidney disease
CRP: C-reactive protein
CTLA-4: cytotoxic T-lymphocyte–associated protein 4
DPN: diabetic peripheral neuropathy
ECM: extracellular matrix
FFA: free fatty acid
H&E stain: hematoxylin and eosin stain
IAPP: islet amyloid polypeptide
IFN: interferon
IL: interleukin
IGF: insulin-like growth factor
IPEX: immunodysregulation polyendrocrinopathy enteropathy X-linked syndrome
MHC: major histocompatibility complex
MMP: matrix metallopeptidase
NO: nitric oxide
NOD: nonobese diabetic
RAGE: receptor for advanced glycation endproduct
ROS: reactive oxygen species
STAT: signal transducer and activator of transcription
T1DM: type 1 diabetes
TCR: T-cell receptor
TLR: toll-like receptor
TNF: tumor necrosis factor
Treg cells: T regulatory cell
